# Medicaid and Medicare Utilization of Direct-Acting Antiviral Medications for Patients With Hepatitis C

**DOI:** 10.1016/j.gastha.2024.10.024

**Published:** 2024-11-04

**Authors:** Xiaohan Ying, Alexander Zhao, Nicole Ng, Russell Rosenblatt, Catherine Lucero, Arun B. Jesudian

**Affiliations:** 1 Department of Internal Medicine, Weill Cornell Medicine, New York, New York; 2 Division of Gastroenterology and Hepatology, Weill Cornell Medicine, New York, New York

Hepatitis C virus (HCV) infection is among the leading causes of liver disease and mortality, with over 2 million people in the United States currently infected.^1^ The approval of direct-acting antivirals (DAAs) has revolutionized its treatment, with estimated cure rates of 97%–99% combined with a superior safety profile compared to previous treatments.^2^^,^^3^

However, adoption of DAA therapy for HCV has been limited by cost, as much as $84,000 for a full treatment.^4^ This has therefore impaired access to HCV treatment for many infected individuals and caused significant economic strain on the US health-care system.^5^^,^^6^ In 2019, generic versions of sofosbuvir-velpatasvir (Epclusa) and ledipasvir-sofosbuvir (Harvoni) were approved by the Food and Drug Administration. In this study, we aim to analyze Medicaid and Medicare Part D utilization and spending on oral HCV medications, and to estimate cost savings associated with the approval of generic DAAs.

In this cross-sectional study, we used publicly available Medicaid and Medicare Part D databases^7^^,^^8^ (2013–2021) to determine the number of 30-day prescriptions and annual spending on DAAs for HCV. Cost per prescription was calculated by dividing annual spending on a specific DAA by the number of 30-day prescriptions. Microsoft Excel (Microsoft, Version 2023) was used for data analysis. JoinPoint Regression Program (version 4.9.1.0; National Cancer Institute, Bethesda, MD) was used for annual percent change (APC) and trends analysis. Cost savings were estimated by multiplying per-prescription savings by the number of 30-day fills for each generic medication.

Eight medications were included in our study: Solvaldi, Harvoni, Epclusa, Zepatir, Mavyret, Vosevi, generic ledipasvir-sofosbuvir, and generic sofosbuvir-velpatasvir. Estimated per-day pricing as well as average wholesale price using UpToDate are included in Table A1.

Between 2013 and 2021, total Medicare spending on DAAs was $31.4 billion; $17.5 billion was spent on Harvoni (55.9%), followed by Sovaldi (17.9%) and Epclusa (16.1%). In 2021 alone however, Epclusa accounted for 62% of total spending, followed by Mavyret (17.2%) and Harvoni (9.5%). Annual spending increased dramatically between 2013 and 2015, followed by steady decline in the ensuing years (2013–2015: APC 1,789, 95% confidence interval [CI]: 1258–2492, *P* < .001; 2015–2021: APC −32.4, 95% CI: −36 to −29, *P* < .001). Figure A.

**Figure:**
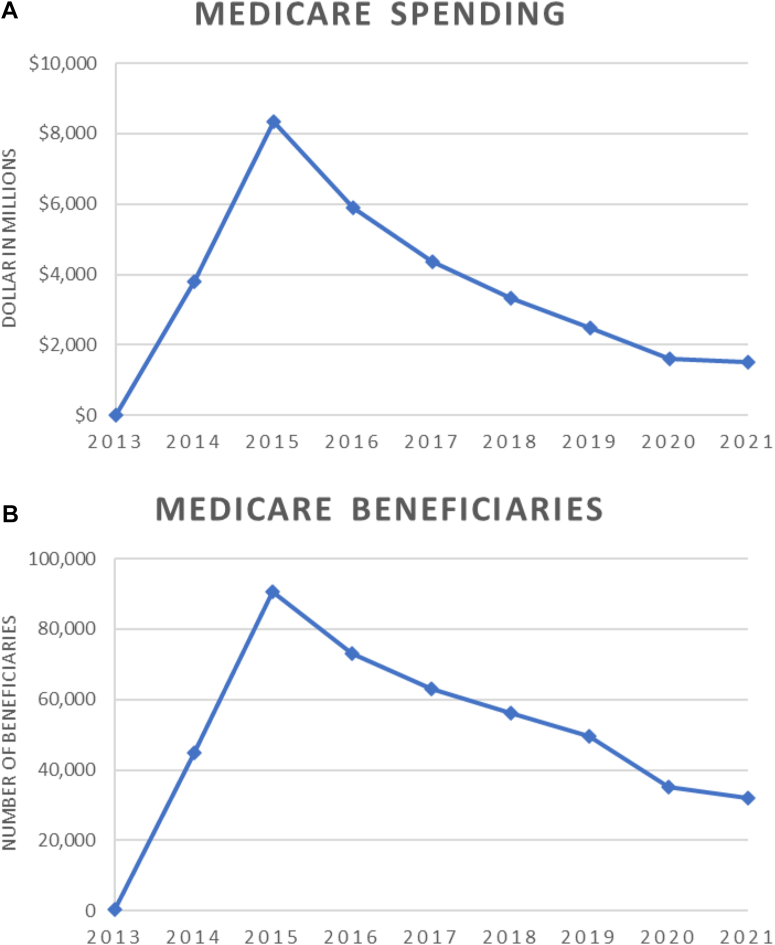

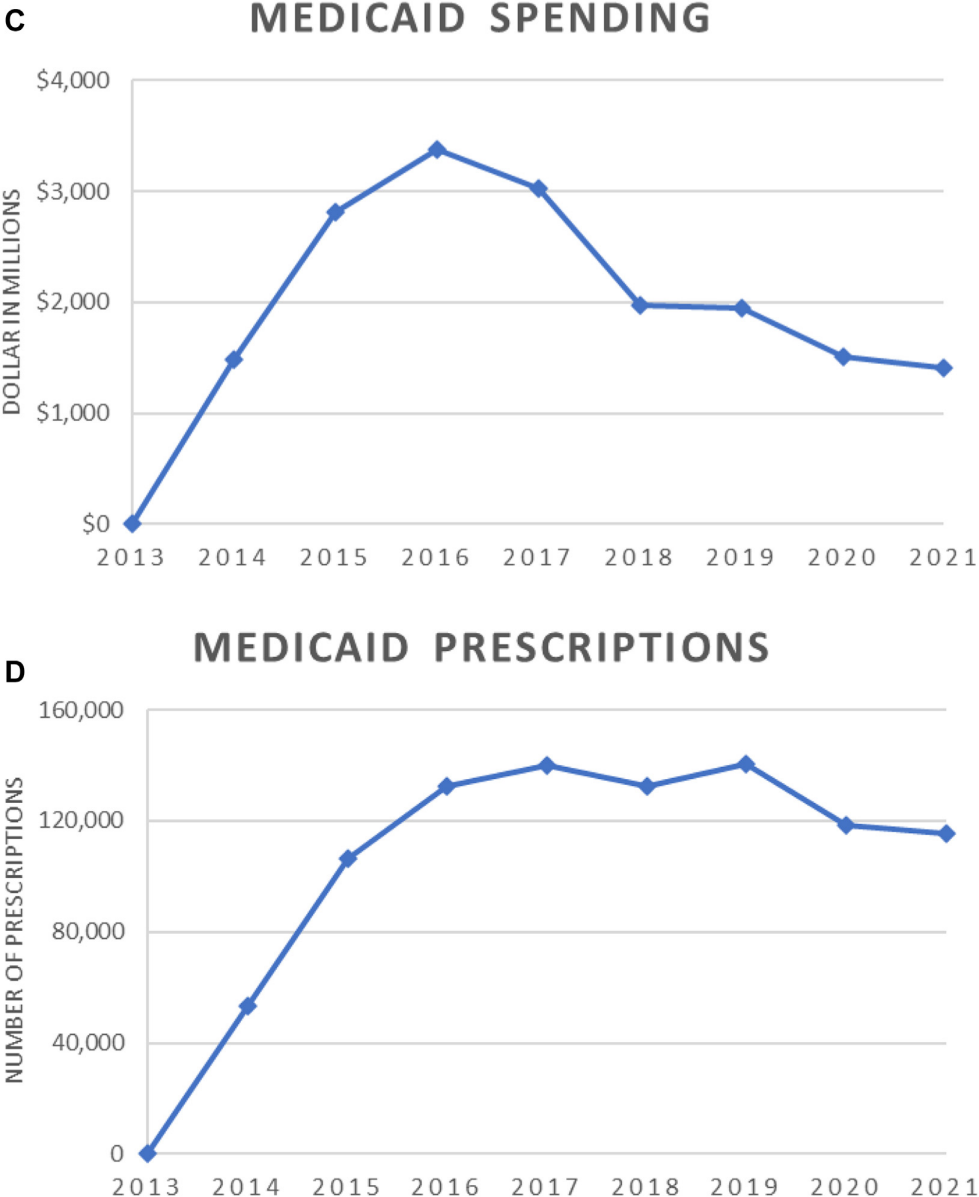
Trends in medicare and medicaid spending and utilization on DAA medications, 2013–2021. (A) Medicare spending on DAA medications. (B) Medicare beneficiaries receiving DAA medications. (C) Medicaid spending on DAA medications. (D) Medicaid prescriptions of DAA medications.

Over the same time period, ∼1.2 million total prescriptions accounting for almost 450,000 Medicare beneficiaries were covered by Medicare; with ∼200,000 people (47.1%) receiving Harvoni, 77,000 (17.4%) receiving Epclusa, and 61,000 (13.8%) receiving Sovaldi. In 2021, 44.5% of patients took Epclusa, followed by Mavyret (30.6%) and generic sofosbuvir-velpatasvir (14.8%). Similar trends were observed in terms of number of beneficiaries receiving DAAs (2013–2015: APC 1,033, 95% CI: 763–1387, *P* < .001; 2015–2021: APC −21.9, 95% CI: −25.4 to −18.2, *P* < .001). Figure B.

Between 2013 and 2021, Medicaid spent $17.5 billion on DAAs, covering 950,000 total prescriptions. In terms of total spending, $6.1 billion was on Harvoni (33.2%), followed by Mavyret (22.2%) and Epclusa (16.5%). Additionally, 336,000 prescriptions (31.9%) were of Mavyret, followed by Harvoni (21.2%) and generic sofosbuvir-velpatasvir (16.5%). In 2021, 48.5% of the spending was on Mavyret covering 44.3% of total prescriptions, and 28.5% of spending was on generic sofosbuvir-velpatasvir covering 43.0% of total prescriptions. Similar trends were observed for total spending and number of prescriptions when compared to Medicare. Figure C and D.

Generic versions of Harvoni (ledipasvir-sofosbuvir) and Epclusa (sofosbuvir-velpatasvir) were both approved by the Food and Drug Administration in 2019. Between 2019 and 2021, generic ledipasvir-sofosbuvir accounted for 5% and 18% of Medicare and Medicaid spending on all ledipasvir-sofosbuvir equivalent (Harvoni, generic), respectively, and covered 12.2% of Medicare prescriptions and 35.4% of Medicaid prescriptions (Table).

**Table:** Medicare and Medicaid Spending/Utilization of Direct-Acting Antiviral Medications for HCV, 2013–2021

Medicare											
Spending ($ in millions)											
	2013	2014	2015	2016	2017	2018	2019	2020	2021	Total	
Harvoni (ledipasvir-sofosbuvir)	$-	$700	$7029	$4399	$2551	$1726	$745	$243	$143	$17,534	55.9%
Sovaldi (sofosbuvir)	$14	$3106	$1318	$932	$211	$11	$3	$4	$2	$5603	17.9%
Epclusa (sofosbuvir-velpatasvir)	$-	$-	$-	$378	$940	$896	$1015	$867	$937	$5033	16.1%
Vosevi (sofosbuvir-velpatasvir-Voxilaprevir)	$-	$-	$-	$-	$74	$145	$90	$67	$58	$434	1.4%
Mavyret (glecaprevir-pibrentasvir)	$-	$-	$-	$-	$71	$461	$539	$296	$260	$1626	5.2%
Zepatier (grazoprevir)	$-	$-	$-	$198	$519	$83	$7	$1	$1	$808	2.6%
Ledipasvir-sofosbuvir	$-	$-	$-	$-	$-	$-	$26	$19	$14	$59	0.2%
Sofosbuvir-velpatasvir	$-	$-	$-	$-	$-	$-	$50	$108	$95	$253	0.8%
Total	$14	$3806	$8347	$5907	$4366	$3321	$2474	$1606	$1510	$31,351	

30 d prescriptions											
	2013	2014	2015	2016	2017	2018	2019	2020	2021	Total	
Harvoni (ledipasvir-sofosbuvir)		21,924	226,251	141,793	81,896	54,605	23,129	7585	4304	561,487	47.5%
Sovaldi (sofosbuvir)	499	109,906	47,220	33,358	7475	389	101	154	86	199,187	16.8%
Epclusa (sofosbuvir-velpatasvir)				15,174	37,671	36,859	41,754	35,165	37,114	203,737	17.2%
Vosevi (sofosbuvir-velpatasvir-Voxilaprevir)					3000	5814	3555	2647	2240	17,255	1.5%
Mavyret (glecaprevir-Pibrentasvir)					5324	34,426	39,925	21,862	18,814	120,351	10.2%
Zepatier (grazoprevir)				10,883	28,177	4767	907	156	82	44,972	3.8%
Ledipasvir-sofosbuvir							2135	1597	1129	4861	0.4%
Sofosbuvir-velpatasvir							6181	13,213	11,323	30,717	2.6%
Total	499	131,830	273,471	201,208	163,543	136,860	117,687	82,377	75,092	1,182,567	

Medicaid											
Spending ($ in millions)											
	2013	2014	2015	2016	2017	2018	2019	2020	2021	Total	
Harvoni (ledipasvir-sofosbuvir)	$-	$95	$2195	$2211	$1220	$316	$120	$42	$27	$6226	35.5%
Sovaldi (sofosbuvir)	$4	$1386	$618	$601	$37	$3	$2	$2	$2	$2654	15.1%
Epclusa (sofosbuvir-velpatasvir)	$-	$-	$-	$342	$938	$491	$536	$389	$262	$2957	16.8%
Vosevi (sofosbuvir-velpatasvir-voxilaprevir)	$-	$-	$-	$-	$24	$70	$60	$54	$52	$260	1.5%
Mavyret (Glecaprevir-Pibrentasvir)	$-	$-	$-	$-	$115	$1026	$1012	$652	$659	$3463	19.7%
Zepatier (Grazoprevir)	$-	$-	$-	$222	$698	$73	$10	$9	$5	$1016	5.8%
Ledipasvir-sofosbuvir	$-	$-	$-	$-	$-	$-	$12	$13	$12	$38	0.2%
Sofosbuvir-velpatasvir	$-	$-	$-	$-	$-	$-	$197	$349	$389	$936	5.3%
Total	$4	$1481	$2813	$3375	$3030	$1978	$1949	$1510	$1408	$17,548	100.0%

Total prescriptions											
	2013	2014	2015	2016	2017	2018	2019	2020	2021	Total	
Harvoni (ledipasvir-sofosbuvir)		3039	79,859	80,340	44,352	11,283	4142	1396	874	225,285	23.6%
Sovaldi (sofosbuvir)	144	50,377	27,223	25,169	1493	133	74	73	67	104,753	11.0%
Epclusa (sofosbuvir-velpatasvir)				15,161	42,967	23,656	24,955	17,107	11,437	135,283	14.2%
Vosevi (sofosbuvir-velpatasvir-voxilaprevir)					1054	3470	2633	2337	2177	11,671	1.2%
Mavyret (glecaprevir-pibrentasvir)					9738	90,596	81,440	51,549	51,500	284,823	29.9%
Zepatier (grazoprevir)				14,363	41,992	4473	1831	2123	1187	65,969	6.9%
Ledipasvir-sofosbuvir							1071	1140	1073	3284	0.3%
Sofosbuvir-velpatasvir							27,112	45,544	50,115	122,771	12.9%
Total	144	53,416	107,082	135,033	141,596	133,611	143,258	121,269	118,430	953,839	100.0%

Similarly, generic sofosbuvir-velpatasvir accounted for 8.2% and 44.4% of Medicare and Medicaid spending on all sofosbuvir-velpatasvir equivalent (Epclusa, generic), respectively, and covered 22.2% of Medicare prescriptions and 69.9% of Medicaid prescriptions.

The introduction of generic medications is estimated to have saved Medicare ∼$1 billion between 2019 and 2021 with the current utilization of generic medications. However, if Medicare were to have utilized generic DAAs at the same rate as Medicaid, it would have saved an additional $1.5 billion. Lastly, we estimate that if Medicare were to only utilize generic medications, it could save an additional $1.5 billion over the course of 3 years.

Since the approval of the first DAAs in 2013, Medicare and Medicaid have spent almost $50 billion on HCV medications. Despite their considerable upfront cost, DAA therapies remain cost effective by reducing mortality and morbidity attributed to advanced liver disease.^7^ However, studies have found that patients often do not receive timely HCV treatment following diagnosis, a phenomenon frequently related to delayed or denied insurance authorization and prohibitive costs associated with copayments and deductibles.^8^

We estimated that generic DAAs, while still relatively underutilized, saved Medicare ∼$1 billion between 2019 and 2021. As the Centers for Disease Control and Prevention aims to eliminate viral hepatitis as a public health threat by 2030, it is crucial to address the underutilization of generic DAA as a means of increasing access to safe and effective HCV therapy.^9^^,^^10^ Different stakeholders (policy makers, prescribers, patients, payors, etc.) must be involved to increase generic medication utilization. For example, 19 states mandate generic substitutions while the other 31 states allow pharmacists to perform generic substitutions.^11^ Expansion of generic substitution would likely increase utilization of generic medication and further reduce spending in the US.

This study has several limitations. First, spending was calculated from Medicare and Medicaid claims data, and does not reflect rebates, coupons, or out-of-pocket costs. Our results are therefore an overestimation of the total Medicare and Medicaid spending on HCV medications, as well as the potential cost savings associated with the use of generic medications. Medicaid has statutory 23.1% rebate for branded and 13% rebate for generic medications, as well as state negotiated rebates; Medicare reported an average of 17.5% rebate for branded medications. Some studies have found that Medicaid rebate accounted for over 50% of its total drug spending.^12^ However, it is not possible to determine actual net spending for HCV drugs as rebate data are proprietary and not publicly available.

In conclusion, Medicare and Medicaid have spent almost $50 billion between 2013 and 2021 on DAAs. Compared to Medicaid, Medicare has underutilized generic DAAs since their approval in 2019. Initiatives to increase generic medication adoption are needed to optimize the number HCV patients treated in a cost-effective manner. Additional studies are warranted to better understand prescription and insurance coverage of generic and branded DAAs, as well as out-of-pocket impacts for patients.

## Supplementary materials

Supplemental Table 1 Per Day Cost of Direct-Acting Antiviral Medications for HCV.
